# A pilot study on spoilage dynamics in industrially produced *Paneer Shahri*, a traditional Iranian fresh cheese

**DOI:** 10.3389/fmicb.2026.1783811

**Published:** 2026-03-26

**Authors:** Delara Moradi Mirhesari, Elham Babaali, Nastaran Nikjoo, Seyed Shahram Shekarforoush, Mohammad Hadi Eskandari, Samane Rahmdel

**Affiliations:** 1 Department of Food Hygiene and Quality Control, School of Nutrition and Food Sciences, Shiraz University of Medical Sciences, Shiraz, Iran; 2 Department of Food Hygiene and Public Health, School of Veterinary Medicine, Shiraz University, Shiraz, Iran; 3 Department of Food Science and Technology, School of Agriculture, Shiraz University, Shiraz, Iran; 4 Microbial Genetics, Interfaculty Institute of Microbiology and Infection Medicine Tübingen (IMIT), University of Tübingen, Tübingen, Germany

**Keywords:** fresh cheese, lactic acid bacteria, microbial spoilage, *Paneer Shahri*, proteolysis

## Abstract

**Introduction:**

Paneer Shahri is a traditional Iranian fresh cheese with high moisture and near-neutral pH, making it susceptible to microbial spoilage. This study aimed to assess spoilage-related changes in Paneer Shahri during storage.

**Methods:**

Microbial, physicochemical, textural, and sensory changes in industrially produced, enzyme-coagulated Paneer Shahri were evaluated during cold storage (8 °C) over 34 days.

**Results:**

Despite pasteurization, spoilage was primarily driven by non-starter lactic acid bacteria (NSLAB), including Lactococcus lactis, Enterococcus faecalis, and Lacticaseibacillus rhamnosus. These bacteria, all proteolytic and psychrotrophic, were consistently recovered throughout storage and became dominant members of the microbial community. Fermentation of residual lactose by LAB led to a significant decline in pH and an increase in titratable acidity, creating acidic conditions that further supported LAB persistence. Proteolysis contributed to texture softening, as reflected by declining chewiness, gumminess, springiness, and cohesiveness. Biogenic amine production by E. faecalis, particularly tyramine, was observed, posing a potential safety concern during prolonged storage. No Enterobacteriaceae, coagulase-positive staphylococci, or aerobic spore-formers were detected, and lipolysis remained negligible throughout the storage period. Yeasts and molds appeared only in the late stages of storage, and sensory analysis revealed declining smell and overall acceptability, consistent with microbial and biochemical spoilage markers.

**Discussion:**

These findings highlight the metabolic activity and persistence of NSLAB as key drivers of spoilage in the absence of classical pathogens, emphasizing the importance of monitoring LAB activity to extend shelf life in fresh high-moisture cheeses.

## 1 Introduction

*Paneer Shahri* is a traditional Iranian fresh cheese that originates from the Fars province. It is typically produced by acidifying milk with vinegar or yogurt and subsequently stored in brine under cool conditions (Supplementary Figure S1A). The name *Shahri*, meaning “urban,” refers to its origin in the city of Shiraz, and distinguishes it from other traditional cheeses that are typically produced in rural areas.

Until recently, this cheese was produced predominantly at household or small-scale levels using traditional acidification techniques. Due to its popularity among consumers, local dairy manufacturers have industrialized the production process using enzymatic coagulation to reproduce the characteristic taste and texture of the traditional product under controlled manufacturing conditions. In the industrial process, milk is concentrated by ultrafiltration, followed by enzymatic coagulation without the use of starter cultures. The final product is stored and distributed under cold chain conditions, as it is classified as a fresh cheese (Supplementary Figure S1B).

Currently, industrial production of *Paneer Shahri* is limited to two manufacturers, both applying highly similar ultrafiltration and enzymatic coagulation protocols without the use of starter cultures. The cheese investigated in this study was obtained from one of these producers and is representative of the product available on the market.

However, like other fresh soft cheeses, *Paneer Shahri* has a high moisture content and relatively high pH, both of which favor rapid microbial growth and spoilage. The absence of protective starter cultures further increases the risk of undesirable microbial growth during storage (Fox et al., 2016).

Two key stages in the production chain, thermal treatment and cold storage, play a critical role in shaping the microbial community associated with spoilage. Despite the pasteurization of raw milk, a critical control point aimed at reducing overall microbial load, this step cannot fully eliminate heat-resistant (thermoduric) microorganisms. These surviving bacteria may persist in the cheese matrix and remain viable throughout processing. In refrigerated dairy products such as fresh cheeses, spoilage is often driven by psychrotrophic bacteria capable of growing at low temperatures and producing proteolytic and/or lipolytic enzymes (Júnior et al., 2018). Moreover, some microorganisms may exhibit both thermoduric and psychrotrophic traits, allowing them to survive pasteurization and subsequently proliferate during cold storage (Ribeiro-Júnior et al., 2025). Nevertheless, the potential contribution of post-pasteurization contamination to the establishment of spoilage-associated microorganisms cannot be ruled out.

Although such spoilage dynamics have been documented in other fresh soft cheeses (Sattin et al., 2016; Murugesan et al., 2018; Kamimura et al., 2019; Marino et al., 2019), no systematic study has yet characterized the spoilage profile of industrially produced *Paneer Shahri*. National standards (Iran National Standards Organization [INSO], 2015, 2024) for this category of cheese specify microbiological limits for hygiene indicators, such as total coliforms, as well as key pathogens, including *Escherichia coli*, coagulase-positive staphylococci, and *Salmonella*, in addition to broad physicochemical thresholds such as a pH of ≤6.6 and moisture content of up to 65% (Table 1).

**Table 1:** Physicochemical and microbiological criteria for fresh cheese without starter cultures based on Iranian national standards (Iran National Standards Organization [INSO], 2015, 2024).

Property	Limit
Chemical properties	
Titratable acidity (g lactic acid/100 g)	≤0.4
pH	≤6.6
Salt (g/100 g)	≤2.5
Moisture (g/100 g)	≤65
Protein (g/100 g)	≥12
Fat content (g/100 g)	≥10 to <25
Microbiological properties	
Total coliforms (CFU/g)	≤10
*Escherichia coli* (per g)	Negative
Coagulase-positive staphylococci (per g)	Negative
Yeasts and molds (CFU/g)	≤10^2^
*Salmonella* (per 25 g)	Negative

In this context, gaining insight into the physicochemical changes, microbial succession, and associated spoilage indicators during storage is essential for improving shelf-life management and ensuring product safety. Therefore, the present study aimed to systematically characterize the spoilage profile of commercially produced enzyme-coagulated *Paneer Shahri* during cold storage (8 °C).

## 2 Materials and methods

### 2.1 Cheese sample preparation and storage

A commercially manufactured, enzyme-coagulated fresh cheese (*Paneer Shahri*), produced by a local dairy facility during the summer season, was subjected to spoilage profiling under cold storage conditions. The cheese was produced using standard industrial procedures from bovine milk (3.2% fat, 8.2% solids-not-fat). Briefly, the milk was pasteurized at 72 °C for 15 s and concentrated by ultrafiltration at 45–50 °C to obtain a retentate containing approximately 12% protein and 15% fat. The retentate then underwent secondary pasteurization at 72–75 °C for 15 s. Following thermal treatment, the retentate and rennet were simultaneously dispensed into polypropylene containers (700 μm thickness, 300 mL capacity) to ensure process consistency. The filled containers were transferred to a coagulation tunnel maintained at 40 °C for 20 min. After coagulation, a cellulose-based sheet was placed on the curd surface, salt was applied, and the containers were sealed with aluminum foil under aerobic conditions prior to cold storage (Supplementary Figure S1B).

On the day of production, samples were collected directly from the processing line, transported under cold conditions, and stored at 8 °C for subsequent monitoring. Microbiological and biochemical analyses were performed throughout a 34-day storage period.

### 2.2 Temperature monitoring

Refrigerator and cheese sample temperatures were recorded on days 0, 1, 3, 4, 6, 7, 9, 10, 12, 13, 15, 16, 18, 19, 21, 22, 24, 26, 27, and 34. Refrigerator temperature was measured using a mercury thermometer placed at the front and back of each shelf. Readings were taken after 5 min with the door closed. On each sampling day, the temperature of two cheese samples was measured. One sample was taken from the upper shelf and one from the lower shelf. To account for possible temperature differences within the refrigerator, the sampling position was alternated between the front and back of the shelves on consecutive sampling days. Cheese sample temperature was measured using a calibrated digital thermometer inserted into the center of each cheese sample, and values were recorded once the temperature stabilized. The same two cheese samples were then used for microbiological, physicochemical, textural, and sensory analyses according to the experimental schedule.

### 2.3 Microbiological analyses

#### 2.3.1 Microbial enumeration

Cheese samples were analyzed on days 0, 3, 6, 9, 12, 15, 18, 21, 24, 27, and 34 of storage. On each day, two samples were homogenized with sterile saline at a ratio of 1:10 (w/v), and serial dilutions were prepared. Microbial enumeration was performed in duplicate for each sample. Aerobic mesophilic microorganisms, as well as yeasts and molds, were enumerated using the pour plate method, whereas all other microbial groups were enumerated using the spread plate technique to allow isolation and purification of surface-grown colonies for further analyses. Aerobic mesophilic and psychrotrophic bacteria were counted on Plate Count Agar (PCA; Liofilchem, TE, Italy) supplemented with 1 g/L skim milk, incubated at 30 °C for 72 h and 7 °C for 7 days, respectively (Todaro et al., 2011). Proteolytic bacteria were cultured on PCA containing 10% sterile skim milk and incubated at 21 °C for 72 h (Javidi et al., 2025). For mesophilic rod-shaped lactic acid bacteria (LAB), samples were plated on MRS agar (Merck Co., Darmstadt, Germany) acidified to pH 5.4 with 5 mol/L lactic acid and incubated anaerobically at 30 °C for 72 h (Settanni et al., 2012), as a selective approach to favor the recovery of rod-shaped LAB by limiting the growth of competing microorganisms. Cocci-shaped mesophilic LAB were cultivated on M17 agar (Liofilchem) under the same anaerobic conditions and temperature for 72 h (Gaglio et al., 2019). Staphylococci were detected on Baird–Parker agar after 48 h at 37 °C (Rahmdel et al., 2019), and Enterobacteriaceae were enumerated on Violet Red Bile Glucose Agar (Liofilchem) after 24 h at 37 °C (Todaro et al., 2011). Aerobic spore-formers were recovered on PCA supplemented with 0.2% starch following heat treatment of the dilutions (80 °C, 10 min) and incubation at 30 °C for 5 days (Sattin et al., 2016). Yeasts and molds were enumerated on Yeast Glucose Chloramphenicol (Merck Co.) agar at 25 °C for 3–5 days (Todaro et al., 2011). Distinct microbial colonies obtained from selective media on different sampling days were purified and preserved at −80 °C in their corresponding growth media supplemented with 25% (v/v) glycerol.

#### 2.3.2 Partial biochemical characterization of bacterial isolates

All purified isolates were initially screened using Gram staining, catalase and oxidase tests, and assessment of proteolytic activity to support preliminary classification. Proteolytic activity was assessed by spot inoculation on PCA supplemented with 10% skim milk and incubated at 21 °C for 48–72 h, with the formation of clear halo indicating positive activity. Lipolytic activity was assessed on tryptic soy agar (Merck Co.) containing 1% Tween 20 (Rahmdel et al., 2025). Sugar fermentation profiles were determined in LAPT broth supplemented with 2% (w/v) individual sugars (d-glucose, d-galactose, d-lactose, d-maltose, d-mannose, d-ribose, d-raffinose, and d-sucrose), using bromophenol blue as a pH indicator. Bacterial suspensions were adjusted to approximately 10^6^ CFU/mL, and the color change was monitored every 24 h for 7 days at 37 °C (Lacerda et al., 2005). Tube coagulase tests were performed on suspected staphylococcal isolates using rabbit plasma.

#### 2.3.3 Molecular identification of bacterial isolates

Genomic DNA was extracted as described by Rahmdel et al. (2019). To assess genetic diversity and reduce redundancy among isolates prior to sequencing, Repetitive Extragenic Palindromic (REP)-PCR was performed using the GTG₅ primer (5′-GTGGTGGTGGTGGTG-3′) (Šalomskienė et al., 2015). PCR reactions (25 μL) contained 12.5 μL of 2 × Taq DNA Polymerase Master Mix RED (1.5 mM MgCl₂, Ampliqon, Copenhagen, Denmark), 0.4 μM of the primer, and 4–7 ng of genomic DNA. The amplification protocol included an initial denaturation at 94 °C for 7 min; 30 cycles of 94 °C for 1 min, 40 °C for 1 min, and 65 °C for 8 min; followed by a final extension at 65 °C for 16 min. Amplicons were separated by electrophoresis on 1.2% agarose gels (20 × 15 cm) at 90 V for 3.5 h, stained with Bio-Safe™ Stain (Bio-Rad Laboratories, Hercules, CA, United States), and visualized under UV light. Banding profiles were analyzed using BioNumerics v7.6 (Applied Maths, Sint-Martens-Latem, Belgium). Dendrograms were generated using the unweighted pair group method with arithmetic mean (UPGMA) clustering and the Pearson correlation coefficient, with isolate grouping performed at a similarity threshold of 80%.

For genus-level confirmation of presumptive *Staphylococcus* isolates, PCR amplification of a 370-bp fragment of the *tuf* gene was performed using *Staphylococcus*-specific primers (Rahmdel et al., 2019).

To identify representative isolates from each REP-PCR cluster, distinct profiles were selected for 16S rRNA gene sequencing. PCR amplification was carried out using primers 8F (5′-AGAGTTTGATCATGGCTCAG-3′) and 15R (5′-AAGGAGGTGATCCAACCGCA-3′) (Rahmdel et al., 2019). Each 25 μL reaction contained 12.5 μL of 2 × Taq DNA Polymerase Master Mix RED (1.5 mM MgCl₂; Ampliqon), 0.4 μM of each primer, and 50–100 ng of template DNA. The PCR protocol included an initial denaturation at 94 °C for 2 min, followed by 35 cycles of 94 °C for 30 s, 52 °C for 30 s, and 72 °C for 90 s, with a final extension at 72 °C for 2 min. PCR products were submitted to Eurofins Genomics (Ebersberg, Germany) for Sanger sequencing using primer 8F and 15R. Resulting sequences were compared to reference data in EzBioCloud^1^ and NCBI GenBank^2^ for species-level identification.

#### 2.3.4 Biogenic amine production of bacterial isolates

Biogenic amine production was evaluated following the protocol of Rahmdel et al. (2024). Selected isolates were grown overnight in tryptic soy broth (TSB) or MRS broth, washed, and resuspended in 5 × phosphate-buffered saline (PBS; pH 7.2) supplemented with 10% glucose and 1 mg/mL of individual amino acid substrates (histidine, tyrosine, phenylalanine, and tryptophan), corresponding to the production of histamine, tyramine, phenethylamine, and tryptamine, respectively. After overnight incubation, supernatants were analyzed by reversed-phase high-performance liquid chromatography (RP-HPLC) using a Poroshell 120 EC-C18 column and diode array detection. Peaks were identified by spiking samples with known standards and comparing their retention times and UV spectra. *Staphylococcus epidermidis* O47 and *Enterococcus faecalis* (*E. faecalis*) ATCC 19433 were used as positive controls.

### 2.4 Physicochemical analysis

The pH and titratable acidity (TA) of homogenized cheese samples were evaluated on days 1, 4, 7, 10, 13, 19, 22, 26, and 34 of cold storage. pH was measured using a Metrohm Model 827 pH meter (Metrohm Ltd., Herisau, Switzerland) calibrated with standard buffers (pH 4.0 and 7.0). For TA, 20 g of each sample was diluted with distilled water to a final volume of 250 mL, filtered, and titrated with 0.1 N NaOH using phenolphthalein as an indicator. TA was expressed as the percentage of lactic acid, calculated using the formula: [(Volume of NaOH (mL) × 0.0009 × 100)/Sample weight (g)] (Abdollahzadeh et al., 2018). Moisture, fat, salt content, total volatile nitrogen (TVN), and free fatty acid (FFA) levels were measured on days 1 and 34 of cold storage. Moisture content was determined by drying approximately 3 g of cheese in pre-weighed aluminum dishes using a hot air oven (Memmert, Schwabach, Germany) at 105 ± 1 °C. Fat content was determined using the Van Gulik method (Tondhoush et al., 2024). Salt content was determined using the Volhard method (Neaves and Langridge, 1998). Briefly, 2 g of homogenized sample was mixed with 2 mL of concentrated nitric acid (65%) in a 100 mL flask and heated in a 49 °C water bath for 15 min. After cooling, 25 mL of 0.1 N silver nitrate was added, and the volume was adjusted to 100 mL with distilled water. The mixture was filtered, and 25 mL of the filtrate was titrated with 0.1 N ammonium thiocyanate in the presence of saturated ferric ammonium sulfate until a reddish-brown endpoint appeared. Salt content (% w/w) was calculated as: [(25 – 4 V) × 0.1 × 0.0585 × 100]/W, where *V* is the volume (mL) of thiocyanate used and *W* is the sample weight (g) (Neaves and Langridge, 1998). Total volatile nitrogen (TVN) was determined using a micro-Kjeldahl distillation method (Peco Co., Shiraz, Iran). Briefly, 10 g of homogenized cheese was mixed with 20 mL of distilled water, 2 g of magnesium oxide, boiling stones, and a few drops of paraffin, and then subjected to steam distillation. Volatile bases were collected in 25 mL of 2% boric acid containing methyl red indicator and titrated with 0.1 N HCl. TVN content (mg/100 g) was calculated using the formula: [(mL of HCl × 0.1 × 14 × 100)/sample weight (g)] (Haouet et al., 2009). Free fatty acid (FFA) content was determined by extracting lipids from 10 g of homogenized sample mixed with 6 g of anhydrous sodium sulfate and 60 mL of diethyl ether. The mixture was shaken at 165 rpm for 1 h at room temperature, then filtered. The residue was extracted three additional times with 20 mL diethyl ether. The combined filtrates were titrated with 0.1 N ethanolic KOH using phenolphthalein as an indicator. FFA content was calculated as mg KOH/g of sample using the formula: [(mL of KOH × 0.1 × 56.1 × 100)/sample weight (g)] (Tondhoush et al., 2024).

All physicochemical analyses were performed on two independent samples per time point, each analyzed in duplicate.

### 2.5 Texture profile analysis

Textural properties of the cheese samples, including hardness, adhesiveness, cohesiveness, springiness, gumminess, and chewiness, were evaluated on days 2 and 27 using a CT3 4,500 Texture Analyzer (Brookfield Engineering Laboratories, Stoughton, United States) equipped with a TA41 cylindrical probe (6 mm diameter, 35 mm height). Day 2 was selected to allow the fresh cheese to equilibrate under cold conditions and ensure consistent textural assessment. Measurements were performed at a crosshead speed of 1 mm/s with a penetration depth of 20%. Tests were conducted directly within the original packaging molds (5 × 7 × 11 cm) using two independent samples per time point, with two technical replicates per sample (Golmakani et al., 2019). To enable comparison across parameters with different measurement scales, all raw values were normalized using min–max scaling across the entire dataset, according to the formula: (*X* – *X*_min_)/(*X*_max_ – *X*_min_), where *X* is the raw measurement, and *X*_min_ and *X*_max_ are the minimum and maximum values for that parameter across all samples. The resulting normalized means for each day were then visualized in a radar chart to illustrate changes in texture profile during storage.

### 2.6 Sensory evaluation

Sensory evaluation was performed at six storage intervals (days 1, 10, 16, 22, 27, and 34) by a five-member panel aged 23–35 years, selected to minimize age-related variability in sensory perception. The assessors evaluated samples (30 g) provided in plastic containers. Evaluations were conducted under white lighting at 8–12 °C. Cold water was supplied between samples for palate cleansing. Each panelist scored the samples for color, flavor, mouthfeel, aroma, texture, and overall acceptability using a 5-point hedonic scale, where 1 denoted “very poor” and 5 denoted “excellent” (Abdollahzadeh et al., 2018).

### 2.7 Statistical analysis

Data were analyzed using IBM SPSS Statistics version 26.0 (IBM Corp., Armonk, NY, United States). One-way ANOVA followed by Duncan’s *post hoc* test was used to compare results across different storage days, while the Friedman test was applied for the analysis of sensory evaluation data. The significance level of *p* < 0.05 was considered statistically significant.

## 3 Results

### 3.1 Storage conditions monitoring

Throughout the 34-day storage period, refrigerator temperatures ranged from 5.50 °C to 7.67 °C, with a mean of 6.71 ± 0.59 °C. Cheese sample temperatures varied between 7.13 °C and 8.95 °C, averaging 7.88 ± 0.35 °C. These values reflect cold storage conditions commonly used during commercial distribution and retail sale of *Paneer Shahri* fresh cheese.

### 3.2 Microbial dynamics during storage

Microbiological analyses were conducted over the 34-day storage period to quantify key microbial groups, including aerobic mesophilic bacteria, psychrotrophs, proteolytic microorganisms, LAB, *Staphylococcus* spp., *Enterobacteriaceae*, aerobic spore-forming bacteria, and yeasts and molds. Representative isolates were subjected to phenotypic and molecular identification to identify the dominant species contributing to spoilage development during cold storage.

#### 3.2.1 Microbial counts

##### 3.2.1.1 Total aerobic mesophilic microorganisms

**Figure 1:**
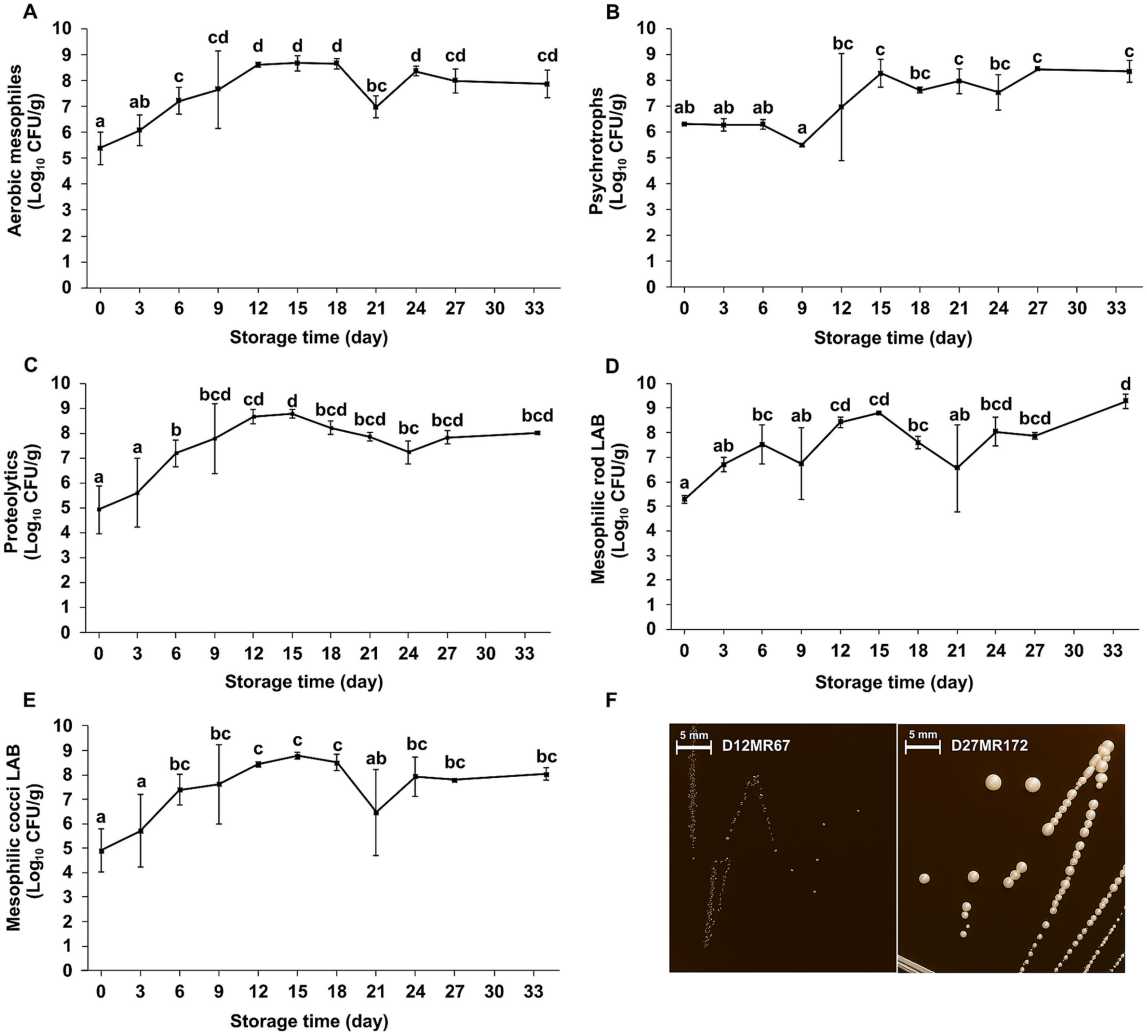
Changes in microbial populations of Iranian fresh cheese (*Paneer Shahri*) during 34 days of cold storage. **(A)** Aerobic mesophilic microorganisms, **(B)** psychrotrophic microorganisms, **(C)** proteolytic microorganisms, **(D)** mesophilic rod-shaped lactic acid bacteria (LAB), **(E)** mesophilic cocci-shaped LAB, and **(F)** morphologically distinct colonies on acidified MRS agar. Values represent mean microbial counts (Log_10_ CFU/g) ± standard deviation from two independent samples tested in duplicate (*n* = 4). Different letters above the data points indicate statistically significant differences between sampling days (*p* < 0.05), based on one-way ANOVA followed by Duncan’s multiple range test.

The total aerobic mesophilic count increased significantly from 5.39 ± 0.64 Log CFU/g on day 0 to 8.61 ± 0.09 Log CFU/g by day 12 (*p* < 0.05), after which it remained at a high level through day 34. A temporary decrease on day 21 (6.98 ± 0.43) was attributed to variability between samples rather than reflecting an overall decline (Figure 1A). A total of 26 representative colonies were isolated from plate count agar and subjected to biochemical characterization. All isolates were Gram-positive and oxidase-negative; 24 were catalase-negative, while 2 were catalase-positive. Proteolytic activity was detected in 18 of the 26 isolates.

##### 3.2.1.2 Psychrotrophic microorganisms

Psychrotrophic counts remained statistically stable during the first 6 days of storage, with values ranging from 6.27 to 6.31 Log CFU/g. A significant increase was observed by day 15, and counts remained statistically unchanged through day 34, reaching 8.34 ± 0.42 Log CFU/g (Figure 1B). A total of 10 isolates were recovered for characterization. All isolates were Gram-positive and oxidase-negative. Catalase activity was detected in two isolates, whereas the remaining eight isolates were catalase-negative. In addition, eight isolates exhibited proteolytic activity.

##### 3.2.1.3 Proteolytic microorganisms

Proteolytic microbial counts increased significantly from 4.92 ± 0.95 Log CFU/g on day 0 to 8.78 ± 0.16 Log CFU/g on day 15 (*p* < 0.05). The increase was significant between days 3 and 6 and continued progressively until day 15. From that time point onward, the counts remained statistically unchanged (Figure 1C). A total of 46 proteolytic isolates were recovered and characterized. All isolates were Gram-positive, catalase-negative, and oxidase-negative.

##### 3.2.1.4 Mesophilic rod-shaped LAB

Mesophilic rod-shaped LAB showed a significant increase over the 34-day storage period, rising from 5.28 ± 0.15 Log CFU/g on day 0 to 9.27 ± 0.30 Log CFU/g on day 34 (*p* < 0.05) (Figure 1D). Two morphologically distinct colony types were consistently observed on acidified MRS agar under anaerobic conditions (Figure 1F). The smaller colony type was dominant throughout storage, whereas the larger colony type was not recovered on days 18, 21, 24, and 34. One representative colony of each type was isolated, when present, at each sampling point for phenotypic characterization. All isolates were Gram-positive, catalase-negative, oxidase-negative, and proteolytically active.

##### 3.2.1.5 Mesophilic cocci-shaped LAB

Mesophilic cocci-shaped LAB increased significantly from 4.91 ± 0.89 Log CFU/g on day 0 to 8.04 ± 0.27 Log CFU/g on day 34 (*p* < 0.05; Figure 1E). A statistically significant increase was detected between the early and later stages of storage, with day 0 differing statistically from most subsequent sampling points. Only a single colony morphology was observed on M17 agar under anaerobic conditions throughout the study period. One representative colony was selected at each time point for purification and subsequent analysis. All isolates were Gram-positive, catalase-negative, oxidase-negative, and proteolytic.

##### 3.2.1.6 *Staphylococcus* spp.

Presumptive *Staphylococcus* isolates were recovered on days 9, 18, and 21, with counts of 3.30 ± 0.26, 3.88 ± 0.53, and 3.47 ± 0.13 Log CFU/g, respectively. Based on biochemical characterization, all isolates were Gram-positive, catalase-positive, and oxidase-negative. PCR amplification targeting the *tuf* gene (370 bp fragment) confirmed their identity as members of the genus *Staphylococcus*. All isolates were coagulase-negative and exhibited proteolytic activity.

##### 3.2.1.7 Other microorganisms

No growth of *Enterobacteriaceae* or aerobic spore-forming bacteria was detected during the entire 34-day storage period. Yeasts and molds were isolated only on days 27 and 34 from one of the two replicates, with counts of 4.24 ± 0.19 and 2.54 ± 0.34 Log CFU/g, respectively.

#### 3.2.2 Identification of spoilage microorganisms

Based on colony morphology and preliminary biochemical characterization, including Gram staining, catalase and oxidase reactions, and proteolytic activity, a total of 46 isolates were selected for further analysis. These isolates included 8 psychrotrophic isolates, 35 proteolytic isolates, 2 mesophilic rod-shaped LAB isolates, and 1 mesophilic cocci-shaped LAB isolate. The selected isolates were considered representative of the dominant microbial populations with potential spoilage activity during cold storage.

##### 3.2.2.1 Sugar fermentation profile of selected isolates

Preliminary identification of the selected microbial isolates included the evaluation of sugar fermentation profiles. All isolates exhibited highly similar fermentation profiles, showing the ability to ferment glucose, galactose, lactose, ribose, mannose, and maltose, while none the isolates were able to ferment rhamnose. Only four isolates, including two mesophilic rod-shaped LAB isolates and two psychrotrophic isolates (D6P53 and D24P174), were unable to ferment sucrose. Due to the high degree of similarity observed in sugar fermentation profiles among the isolates, molecular methods were employed for more accurate differentiation.

##### 3.2.2.2 Molecular identification of selected isolates

To further differentiate isolates exhibiting similar biochemical characteristics, molecular fingerprinting was performed using REP-PCR. Isolates sharing ≥80% similarity in their REP-PCR banding patterns were grouped into clusters. This analysis revealed the presence of seven major clusters, along with eight single-profile isolates that did not cluster with any other isolate. One or more representative isolates from each cluster were selected for 16S rRNA gene sequencing. A 1,500 bp fragment of the 16S rRNA gene was amplified and sequenced. The resulting sequences were compared with reference sequences available in the EzBioCloud and GenBank databases for species-level identification. Overall, 31 isolates, including 1 psychrotrophic isolate, 29 proteolytic isolates, and 1 mesophilic cocci-shaped LAB isolate, were identified as *Lactococcus lactis* (*L. lactis*). Eleven isolates, comprising 5 psychrotrophic isolates and 6 proteolytic isolates, were identified as *E. faecalis*. Two mesophilic rod-shaped LAB isolates were identified as *Lacticaseibacillus rhamnosus* (*Lb. rhamnosus*). In addition, two psychrotrophic isolates were assigned to the genus *Staphylococcus*; however, sequence similarity was insufficient to allow clear discrimination between *S. saprophyticus* and *S. xylosus*.

**Figure 2:**
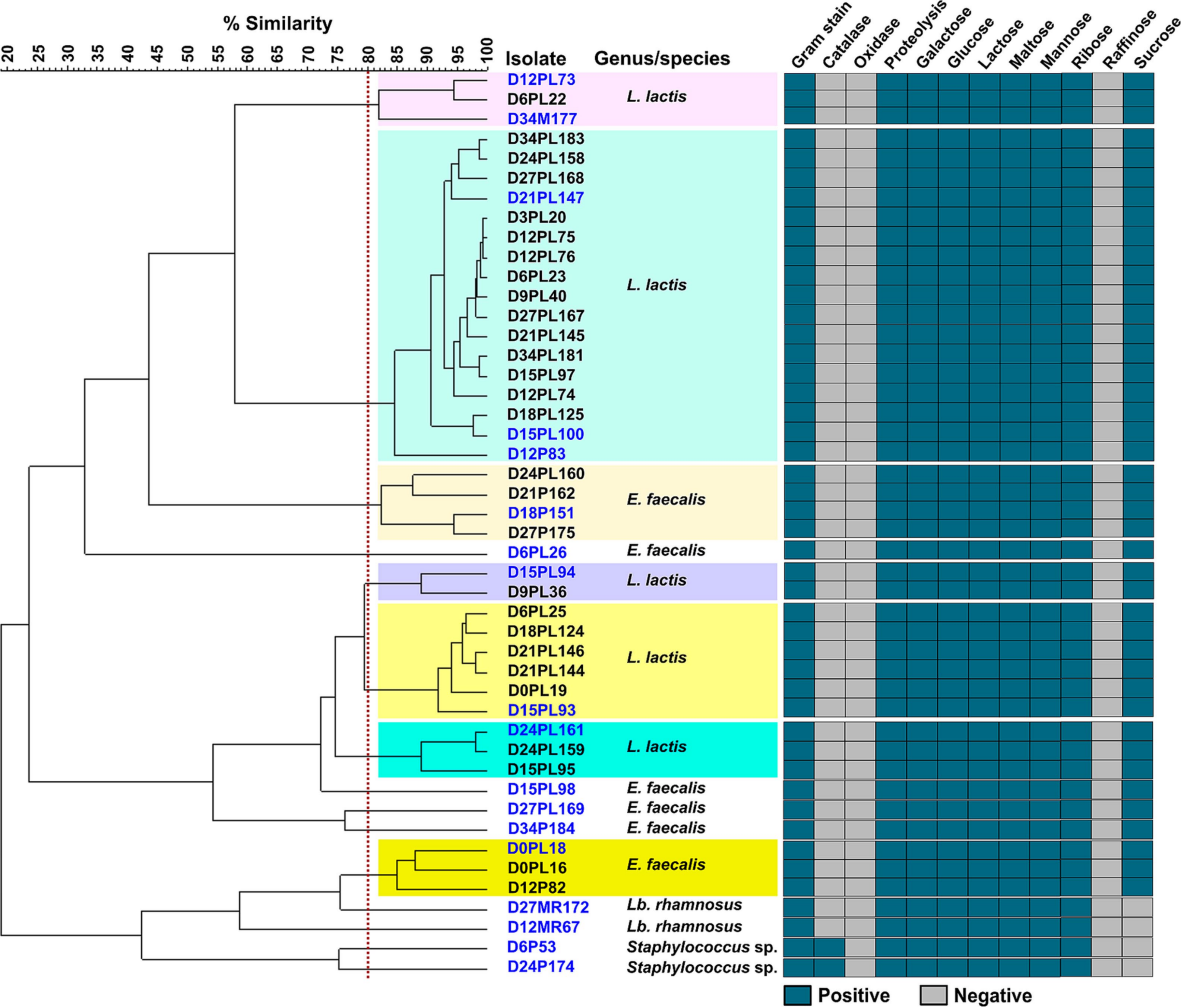
Cluster analysis and phenotypic profiling of 46 microbial isolates recovered from Iranian fresh cheese (*Paneer Shahri*) over 34 days of cold storage. Isolates were grouped based on REP-PCR (Repetitive Extragenic Palindromic-Polymerase Chain Reaction) fingerprinting using the UPGMA (Unweighted Pair Group Method with Arithmetic Mean) algorithm and Pearson correlation coefficient (optimization: 1%, curve smoothing: 0%, negative similarities clipped to zero). Clusters were defined at ≥80% similarity and are indicated by different background colors. Isolates identified to the genus or species level through 16S rRNA gene sequencing are labeled in blue. The accompanying heatmap shows the results of phenotypic tests including Gram staining, catalase and oxidase reactions, proteolytic activity, and carbohydrate fermentation patterns. Each isolate label begins with “D” followed by the day of isolation, then a code representing the isolation medium: P (psychrotrophs), PL (proteolytics), MR (MRS for mesophilic rod LAB), or M (M17 agar for mesophilic cocci LAB). This is followed by the isolate number. *E. faecalis*, *Enterococcus faecalis*; *L. lactis*, *Lactococcus lactis*; *Lb. rhamnosus*, *Lacticaseibacillus rhamnosus*; LAB, lactic acid bacteria.

A summary of REP-PCR-based clustering, phenotypic profiles, and 16S rRNA gene-based identification of the 46 selected isolates is presented in Figure 2.

##### 3.2.2.3 Biogenic amine production and lipolytic activity by selected isolates

**Figure 3:**
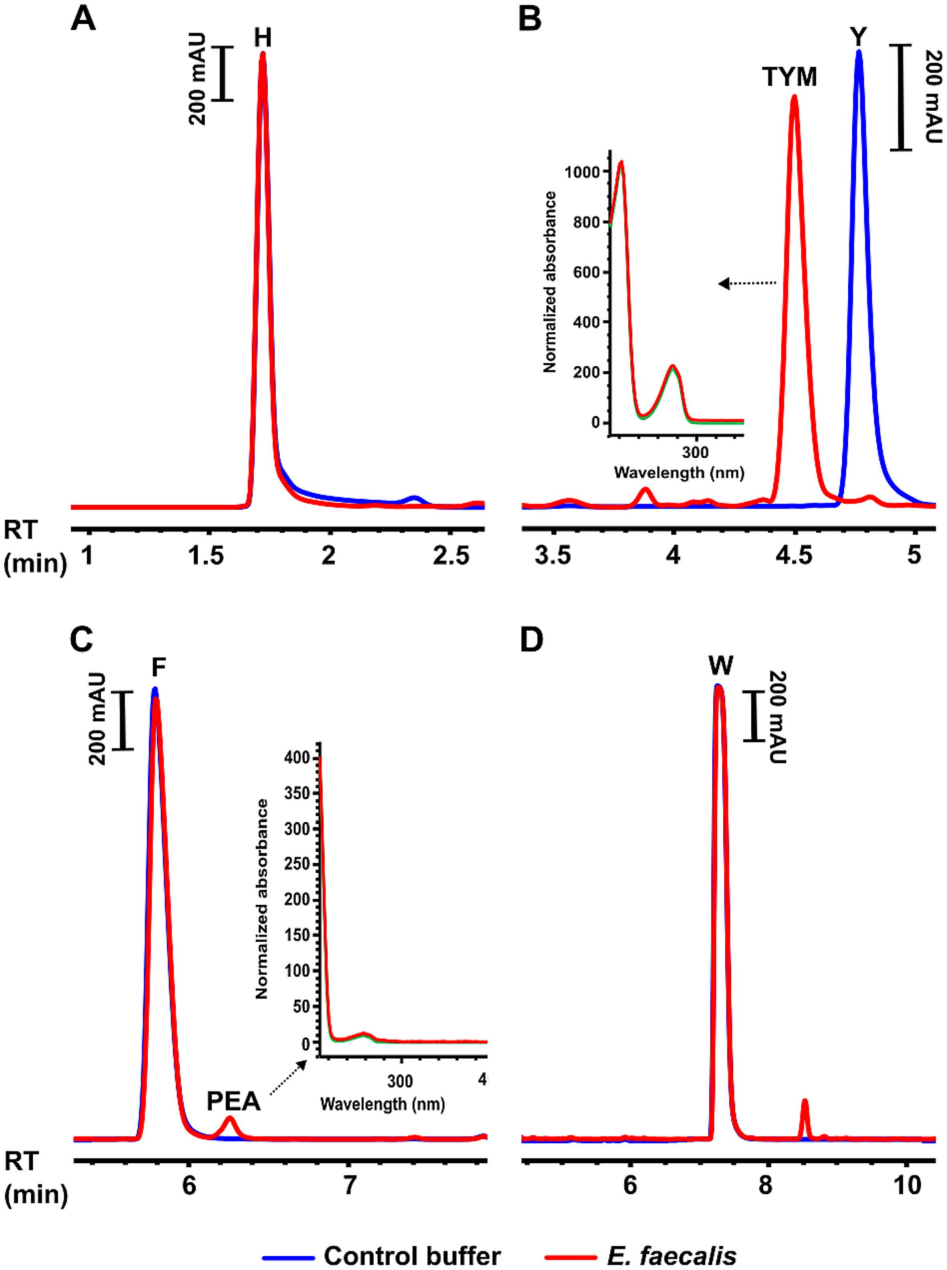
Biogenic amine production by *Enterococcus faecalis* isolate D15PL98 from Iranian fresh cheese (*Paneer Shahri*). Reversed-phase HPLC chromatograms showing decarboxylation activity of *E. faecalis* D15PL98 against individual amino acid substrates after overnight incubation in 5 × PBS buffer with 10% glucose (pH 6.8) containing 1 mg/mL of each amino acid. Red traces represent culture supernatants of *E. faecalis* incubated with each amino acid; blue traces show control buffer with amino acids but no bacteria. **(A)** No decarboxylation of histidine (H). **(B)** Tyrosine (Y) decarboxylation resulting in tyramine (TYM) production. **(C)** Phenylalanine (F) decarboxylation producing trace levels of phenylethylamine (PEA). **(D)** No decarboxylation of tryptophan (W). Insets in panels **(B,C)** show UV absorbance spectra of the detected amine peaks (red), overlaid with spectra of authentic standards for TYM and PEA (green), respectively, confirming compound identity. RT, retention time; mAU, milli-absorbance units.

Two isolates of *E. faecalis* (D15PL98 and D18P151), four isolates of *L. lactis* (D12PL73, D15PL93, D21PL147, and D34M177), and two isolates of *Lb. rhamnosus* were tested for decarboxylase activity against histidine, tyrosine, phenylalanine, and tryptophan. Only the *E. faecalis* isolates exhibited detectable decarboxylation activity, producing tyramine and trace levels of phenylethylamine, which was consistent with tyrosine decarboxylase activity (Figure 3).

These selected isolates were also screened for lipolytic activity; however, none of the isolates showed detectable lipase production under the experimental conditions. *S. aureus* JE2 and a nasal isolate of *Cutibacterium acnes* were included as positive controls under aerobic and anaerobic conditions, respectively, and both exhibited clear lipolytic activity.

### 3.3 Chemical spoilage indicators

**Figure 4:**
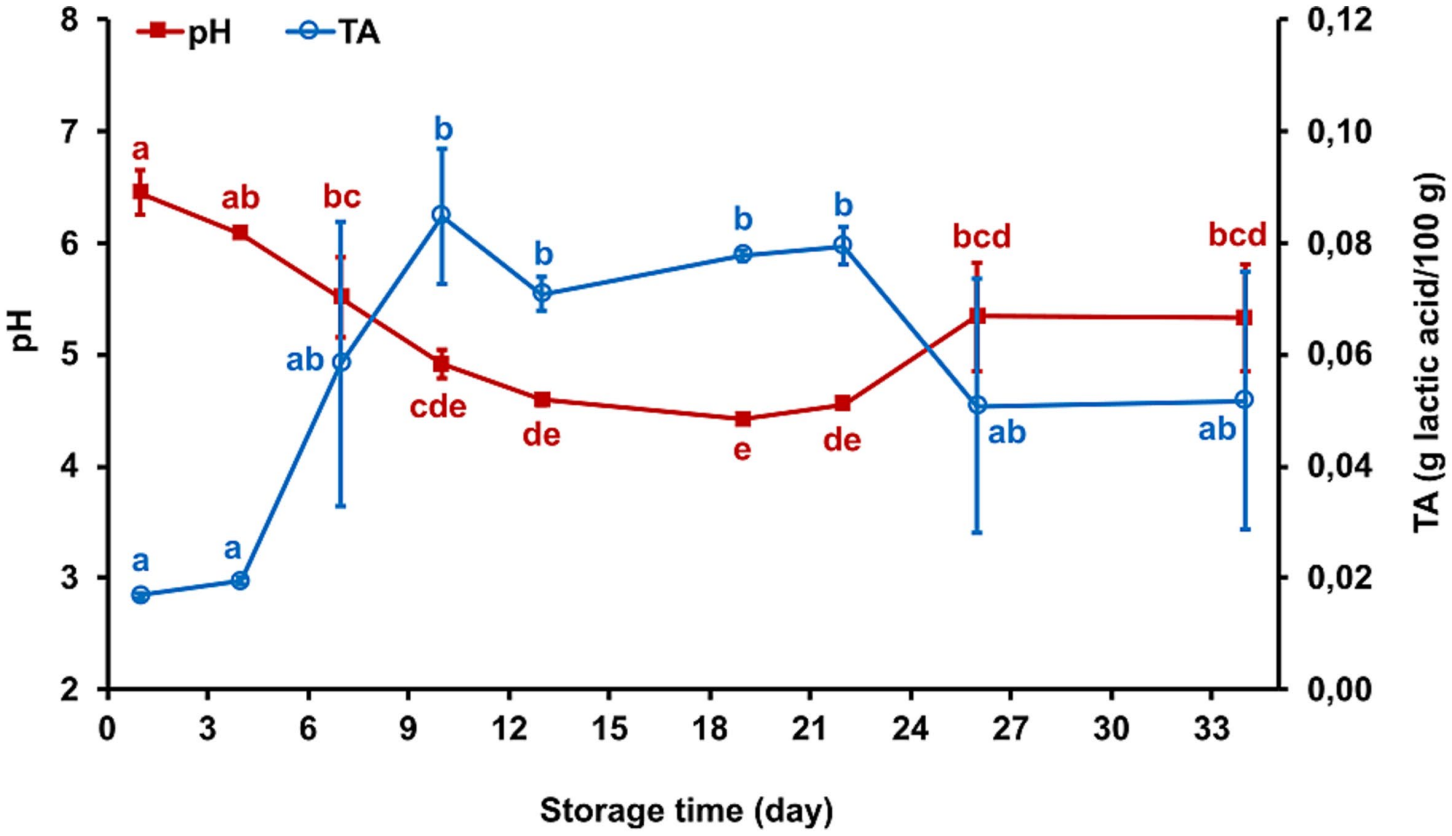
Changes in pH and titratable acidity (TA) of Iranian fresh cheese (*Paneer Shahri*) during 34 days of cold storage. Values represent mean ± standard error from two independent samples tested in duplicate (*n* = 4). Different letters at each time point indicate statistically significant differences (*p* < 0.05) according to Duncan’s multiple range test. TA is expressed as grams of lactic acid per 100 g of cheese.

Significant acidification occurred during storage, with pH decreasing from 6.46 ± 0.20 on day 1 to a minimum value of 4.42 ± 0.01 on day 19 (*p* < 0.05). The pH then showed a slight increase, reaching 5.33 ± 0.48 on day 34. Correspondingly, TA increased significantly, from 0.02 ± 0.00 g lactic acid/100 g on day 1 to 0.08 ± 0.01 by day 10 (*p* < 0.05), with the highest values maintained until day 22. A slight decline in TA was observed during the final days of storage, although TA values measured on days 26 and 34 remained elevated compared to initial levels. These trends in pH and TA indicate an initial acidification followed by partial recovery, likely due to microbial activity and proteolysis (Figure 4). As shown in Table 2, the moisture content of the fresh cheese decreased significantly from 71.80 ± 2.11 g/100 g on day 1 to 67.68 ± 1.34 g/100 g on day 34 (*p* = 0.017). No statistically significant changes were observed in salt, fat, and FFA contents over the storage period (*p* > 0.05). TVN, an indicator of protein degradation, increased from 6.57 ± 2.96 mg/100 g to 14.48 ± 6.01 mg/100 g, approaching statistical significance (*p* = 0.056).

**Table 2:** Physicochemical properties of Iranian fresh cheese (*Paneer shahri*) on days 1 and 34 of cold storage (Mean ± SD).

Day	Moisture (g/100 g)	Salt (g/100 g)	Fat (g/100 g)	TVN (mg/100 g)	FFA (mg KOH/g)
1	71.80 ± 2.11	1.81 ± 0.14	16.25 ± 1.77	6.57 ± 2.96	1.06 ± 0.06
34	67.68 ± 1.34	1.85 ± 0.14	15.00 ± 0.00	14.48 ± 6.01	0.66 ± 0.51
*p* value	0.017	0.702	0.423	0.056	0.239

Values represent mean ± standard deviation (SD) from two independent samples tested in duplicate (*n* = 4). Statistical differences between days were determined using one-way ANOVA followed by Duncan’s *post hoc* test. Differences were considered significant at *p* < 0.05. TVN, total volatile nitrogen; FFA, free fatty acid.

### 3.4 Texture profile changes

**Figure 5:**
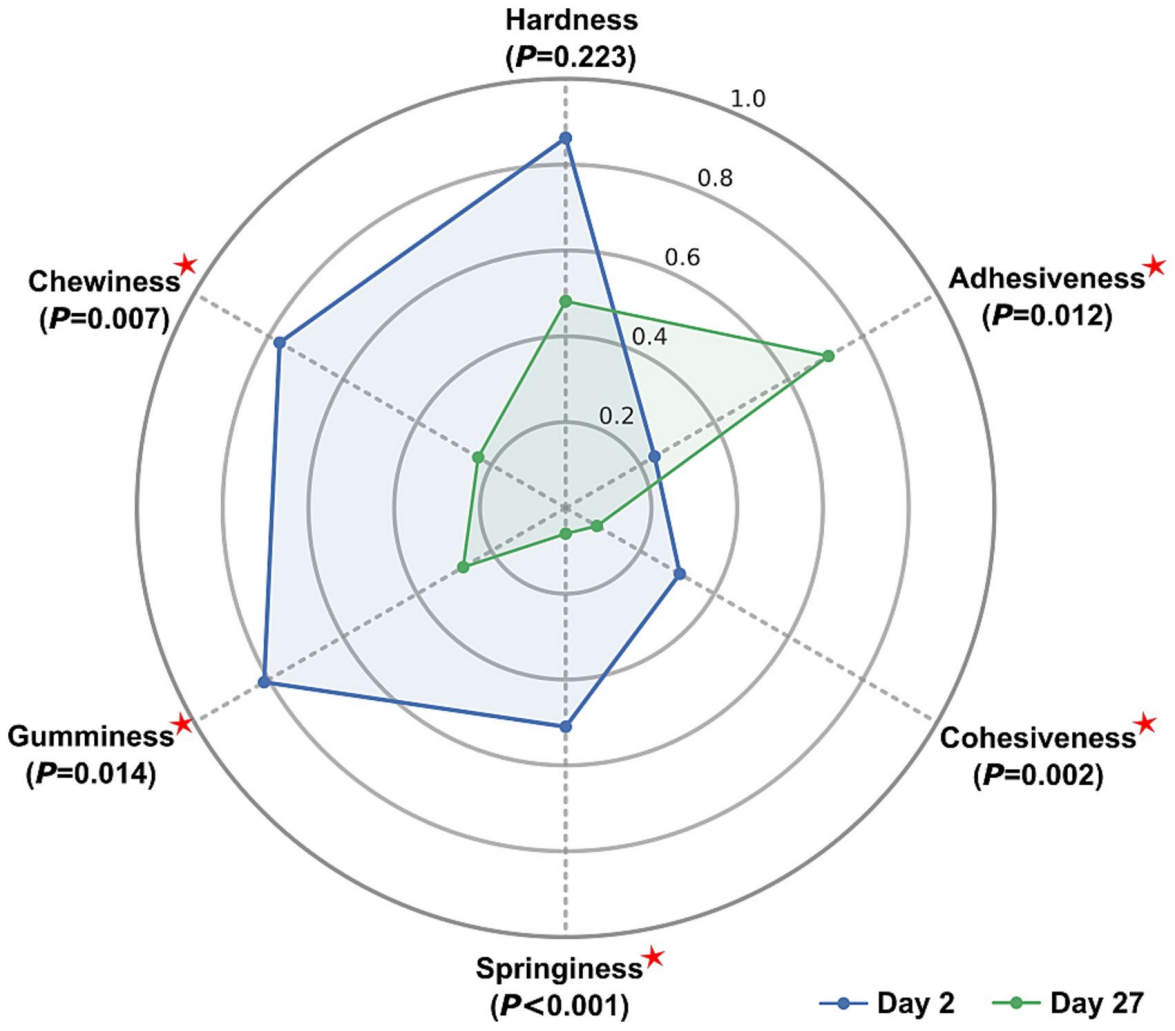
Radar chart of normalized textural attributes (hardness, adhesiveness, cohesiveness, springiness, gumminess, chewiness) of Iranian fresh cheese (*Paneer Shahri*) on day 2 and day 27 of cold storage. Data represent mean values from two independent samples tested in duplicate (*n* = 4), normalized using min–max scaling. Differences between days were assessed using one-way ANOVA followed by Duncan’s *post hoc* test. Red stars (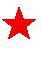) indicate significant differences (*p* < 0.05).

The textural properties of the fresh cheese samples changed noticeably during cold storage (Figure 5). Significant reductions were observed in chewiness, gumminess, springiness, cohesiveness, and adhesiveness between days 2 and 27 (*p* < 0.05), indicating structural degradation and softening of the cheese matrix over time. Although hardness also exhibited a decreasing trend, this change was not statistically significant (*p* = 0.223). These changes are indicative of protein matrix breakdown and moisture redistribution, affecting the cheese texture during storage.

### 3.5 Sensory evaluation

**Figure 6:**
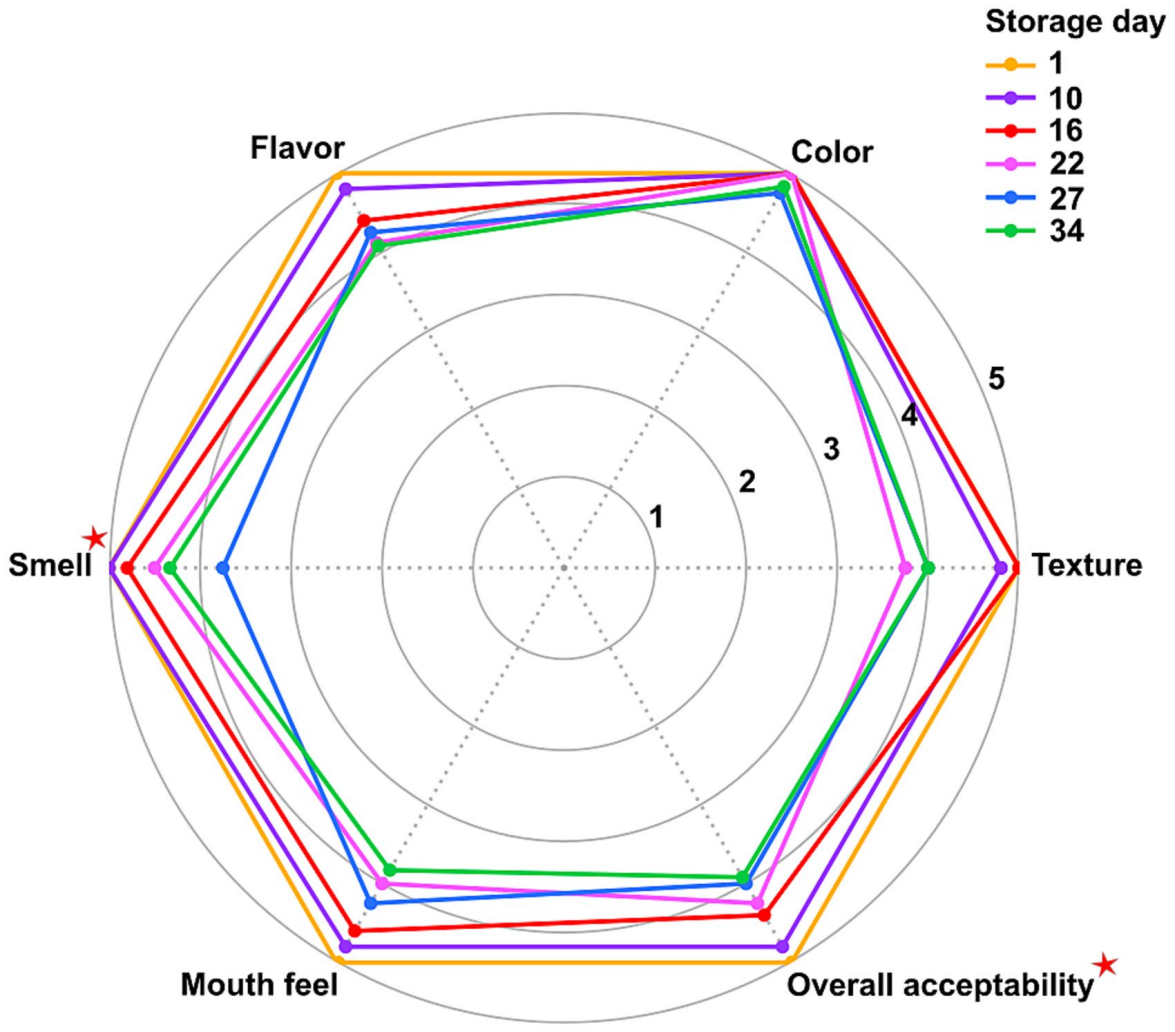
Radar chart of the sensory scores of Iranian fresh cheese (*Paneer Shahri*) over six cold storage intervals (Days 1, 10, 16, 22, 27, and 34). Data represent mean values from two independent samples tested in duplicate (*n* = 4). Sensory attributes were evaluated using a 5-point hedonic scale, where 1 denoted “very poor” and 5 denoted “excellent.” Differences between days were assessed using the Friedman test. Red stars (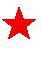) indicate significant differences (*p* < 0.05).

Sensory evaluation of the cheese samples over the 34-day storage period revealed a general decline in all sensory attributes, with the exception of color, which remained relatively stable throughout the storage period (Figure 6). The Friedman test confirmed that the changes observed over time were statistically significant for smell (Exact Sig. = 0.028) and overall acceptability (Exact Sig. = 0.036). Mean scores for smell decreased from 5.00 on day 1 to 3.75 on day 27, while overall acceptability declined from 5.00 to 3.92 by day 34. Although other sensory attributes, including texture, flavor, and mouthfeel, also exhibited decreasing mean scores over time, these changes were not statistically significant (Exact Sig. > 0.05).

## 4 Discussion

Fresh enzyme-coagulated cheeses, including *Paneer Shahri*, are nutrient-rich dairy products with inherently short shelf lives due to their high moisture content, near-neutral pH, and absence of starter cultures. These attributes create an ideal environment for microbial growth and enzymatic spoilage, particularly in the absence of maturation steps (Fox et al., 2016). Throughout storage, the dynamics of microbial succession and the resulting biochemical changes play a critical role in determining product stability and safety.

In this study, microbiological analysis revealed that all isolated microorganisms were Gram-positive, and no coliforms, coagulase-positive staphylococci, or aerobic spore-forming bacteria were detected during the 34-day storage period. The absence of these microbial groups, particularly coagulase-positive *Staphylococcus aureus* and Enterobacteriaceae, is consistent with the use of pasteurized milk and the maintenance of hygienic processing conditions (Touch and Deeth, 2009). Comparable findings have been reported for other high-moisture fresh cheeses, including Mozzarella, Ricotta, and Italian Fruhe, in which the occurrence of classical pathogens was minimal or absent. In those studies, spoilage was predominantly attributed to post-pasteurization contamination and/or the proliferation of spoilage-associated taxa such as coagulase-negative staphylococci, coliforms, or *Pseudomonas* spp., often linked to inadequate environmental hygiene, cross-contamination, or contaminated water systems used during processing (Haouet et al., 2009; Guidone et al., 2016; Murgia et al., 2020).

Yeasts and molds were detected only in the later stages of storage (days 27 and 34), and only in one of the two parallel samples. Their appearance coincided with a notable drop in pH, which may have reduced bacterial competition, thereby enabling opportunistic fungi to proliferate. While most fungal contaminants are typically eliminated during pasteurization, the late-stage detection observed in this study suggests that a small number of heat-resistant fungi may have survived processing and subsequently encountered favorable conditions for growth as acidity increased (Awasti and Anand, 2020; Dash et al., 2022). Although fungal growth remained limited, these findings indicate that prolonged storage may allow fungi to compete more effectively within the microbial community, potentially altering the spoilage dynamics of *Paneer Shahri*.

The dominant spoilage microbiota identified throughout the storage period consisted of non-starter lactic acid bacteria (NSLAB) with proteolytic activity, namely *L. lactis*, *E. faecalis*, and *Lb. rhamnosus*, which were the only species recovered in this study, highlighting their strong ecological fitness within the cheese matrix. NSLAB, though typically representing a minor fraction of the raw milk microbiota, are known for their thermoduric properties, which enable survival through pasteurization, while their psychrotrophic capacity allows them to proliferate under cold conditions (Touch and Deeth, 2009; Zhao et al., 2024). These species exhibit high resilience, with tolerance to a wide range of environmental stresses, including low temperatures (as low as 2 °C), reduced moisture content, elevated salt concentrations (up to 6%), and acidic pH values as low as 4.9. Their enzymatic activities, particularly proteolysis and lactose fermentation, can substantially modify the physicochemical characteristics of cheese during storage (Montel et al., 2014).

The persistent presence of *L. lactis* is particularly noteworthy, as this mesophilic species has frequently been reported as a primary spoilage organism in raw milk and dairy products stored at temperatures ranging from 10 to 37 °C. Its metabolic activity, including the production of lactic acid and, to a lesser extent, acetic and propionic acids, contributes to acidification and sensory deterioration (Touch and Deeth, 2009). In the present study, *L. lactis* appeared to play a central role in the progressive decline in pH, accompanied by a corresponding increase in titratable acidity, findings that are consistent with lactose metabolism and further supported by the observed increase in TVN, indicating ongoing proteolytic degradation during storage.

In parallel, *E. faecalis* isolates were also implicated in spoilage progression due to their proteolytic potential and ability to grow under cold conditions (Terzić-Vidojević et al., 2021). Beyond proteolysis, *E. faecalis* possesses an advanced amino acid metabolism system that enables efficient utilization of nitrogen from casein-derived peptides through extracellular proteases and a wide array of aminopeptidases. Under nutrient-limited and acidic conditions, both of which are relevant during the later stages of cheese storage, this species can activate amino acid decarboxylation pathways (Dapkevicius et al., 2021). In this study, tyrosine decarboxylase activity was detected in the *E. faecalis* isolates, suggesting their involvement in the formation of biogenic amines such as tyramine. This metabolic capability may support cellular survival under stress conditions while simultaneously contributing to flavor deterioration and raising potential safety concerns during extended storage (Dapkevicius et al., 2021).

Similar patterns of NSLAB dominance have been reported in fresh and minimally ripened cheeses produced without starter cultures across various geographic regions, including Zlatar cheese from Serbia (Veljovic et al., 2007), Anevato cheese from Greece (Hatzikamari et al., 1999), Coalho and Marajó cheeses from Brazil (Medeiros et al., 2016; Kamimura et al., 2019), and several Mexican cheese varieties (Murugesan et al., 2018). In these products, *L. lactis*, *E. faecalis*, and species belonging to the genus *Lactobacillus* have frequently been reported as dominant members of the microbial community, reinforcing the concept that NSLAB play a central role in spoilage processes in fresh cheeses manufactured without defined starter cultures.

Textural changes in *Paneer Shahri* during storage appeared to result primarily from microbial activity rather than from compositional variation. Although moisture and fat content are generally recognized as key determinants of cheese texture (Ahmed et al., 2005), the relatively stable levels of moisture and fat observed in *Paneer Shahri* throughout storage suggest that the progressive softening was primarily driven by biochemical activity. In line with this, previous studies have emphasized the central role of protein content and proteolysis in shaping cheese texture, particularly in high-moisture varieties (Kindstedt and Fox, 1993; Eroglu et al., 2016; Rathod et al., 2025). In the present study, the high prevalence of proteolytic isolates supports this interpretation, indicating that active degradation of the casein network was a major contributor to structural weakening. Proteolysis disrupts protein–protein interactions within the cheese matrix, leading to a reduction in hardness, cohesiveness, springiness, and gumminess.

In contrast, adhesiveness increased during storage, which may reflect progressive breakdown of the protein structure and the formation of a softer and less cohesive gel matrix. Furthermore, the gradual decline in pH observed in this study may have further exacerbated the loss of structural integrity, as acidification promotes casein demineralization and destabilization of the protein network. This interaction between proteolysis and acidification is consistent with findings reported by Sant’Ana et al. (2013), who observed that in Minas fresh cheese, proteolytic activity by LAB led to casein hydrolysis and mineral loss, ultimately resulting in reduced hardness and texture softening.

Despite the proteolytic activity detected during storage, lipolysis remained negligible in *Paneer Shahri*, as indicated by the absence of a significant increase in free fatty acid levels. This observation is consistent with the lack of lipolytic activity detected in representative isolates of the dominant LAB species (*L. lactis*, *E. faecalis*, and *Lb. rhamnosus*), indicating that these strains did not actively contribute to fat hydrolysis. Moreover, pasteurization likely inactivated indigenous milk lipases, thereby further limiting lipolytic potential. These findings are in agreement with previous reports suggesting that indigenous milk microflora and NSLAB generally play a limited role in lipolysis, particularly in cheeses produced from pasteurized milk (Hickey et al., 2007).

Sensory evaluation results supported microbiological and biochemical findings, revealing a significant decline in smell and overall acceptability during storage. These changes likely reflect the metabolic activity of spoilage-associated LAB, whose proteolytic and acidifying activities are known to generate off-odors and negatively affect flavor perception. Although changes in texture, flavor, and mouthfeel were not statistically significant, their downward trends were consistent with the observed structural softening and increased adhesiveness. In contrast, color remained relatively stable, suggesting that it was less affected by the spoilage mechanisms active in *Paneer Shahri* during storage.

This study demonstrated that spoilage in *Paneer Shahri* during cold storage was primarily driven by NSLAB, whose proteolytic and acidifying activities contributed to structural degradation and sensory decline, while pathogenic bacteria were absent and lipolysis remained negligible. Although these findings provide valuable insights into the microbial and biochemical processes affecting product quality and shelf life, several limitations should be acknowledged. Only a single production batch from the summer season was evaluated; therefore, potential seasonal or batch-to-batch variability cannot be excluded, even though industrial production is currently limited to two manufacturers applying highly similar processing protocols. In addition, the lack of advanced microbial profiling approaches, such as next-generation sequencing (NGS), may have limited a more comprehensive understanding of microbial community dynamics. Furthermore, although Iranian national standards do not permit the use of preservatives in cheese production, the application of natural antimicrobial systems warrants consideration for high-moisture fresh cheeses such as *Paneer Shahri*. Previous studies have shown that nisin, particularly when used in combination with lysozyme, can exert synergistic inhibitory effects against spoilage-associated lactobacilli (Chung and Hancock, 2000; Sobrino-López and Martín-Belloso, 2008). In addition to antimicrobial compounds, the use of protective cultures composed of selected lactic acid bacteria capable of inhibiting spoilage microorganisms without functioning as preservatives, represents another promising strategy for extending cheese shelf life (Eren-Vapur et al., 2022). The incorporation of such hurdle-based approaches, where legally permissible or evaluated within a research framework, may offer a practical means of mitigating LAB-driven spoilage in fresh cheeses of this type.

Future research should explore seasonal variability in spoilage patterns, evaluate the feasibility and regulatory framework for natural antimicrobial interventions, including protective cultures, and apply advanced molecular techniques to characterize the full microbial community. Together, these approaches could support the development of more effective strategies to extend shelf-life and maintain the quality of high-moisture fresh cheeses, such as *Paneer Shahri*.

## Data Availability

The 16S rRNA sequences determined in this study have been deposited in the GenBank database under accession numbers PZ050344 to PZ050347.

## Footnotes

1
http://www.ezbiocloud.net

2
https://blast.ncbi.nlm.nih.gov
