# Supplementary material for: A pilot study on spoilage dynamics in industrially produced Paneer Shahri, a traditional Iranian fresh cheese
Source: Front Microbiol. 2026 Mar 26;17:1783811. doi: 10.3389/fmicb.2026.1783811 (PMC13062191; doi:10.3389/fmicb.2026.1783811)
Supplement: Supplementary file 1 [file Data_Sheet_1.pdf]

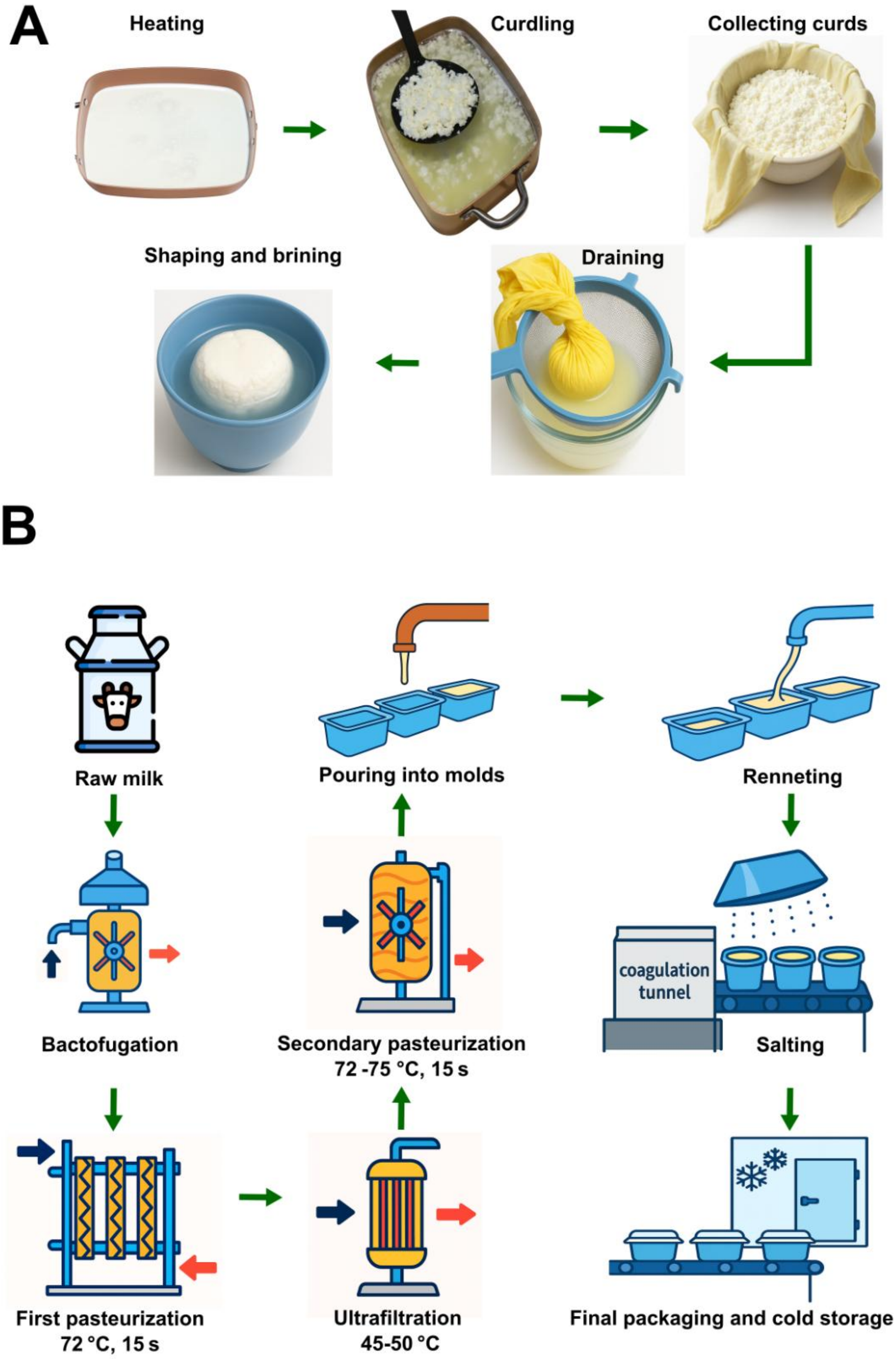

Supplementary Figure 1. Traditional and industrial production of Iranian fresh cheese (Paneer Shahri). **A.** Schematic representation of the traditional method, including heating, curdling, collecting curds, draining, and shaping with brining; **B.** Industrial production flowchart involving pasteurization, ultrafiltration, secondary pasteurization, molding, enzymatic coagulation, salting, and final cold storage.
